# Reducing orthopaedic theatre exposure during the COVID-19 lockdown: does a shift towards virtual reality-based training offer a solution?

**DOI:** 10.1080/17453674.2020.1845437

**Published:** 2020-11-11

**Authors:** Adam Arshad, Amit Zaveri, Henry Atkinson

**Affiliations:** a Department of Emergency Medicine, University College London Hospital;; b North Middlesex University Hospital, London, UK

## Abstract

Orthopaedic training in the United Kingdom has changed little from the Halstedian apprenticeship model of graduated responsibility, with the mantra “see one, do one, teach one”. Whilst still relevant in surgical teaching, the current and ongoing disruption to surgical training secondary to the coronavirus disease 2019 (COVID-19) outbreak highlights the need for alternative methods of experiential surgical learning, which allow for the development of the knowledge, skills, and attitudes of orthopaedic surgeons, to be sought.

Virtual reality-based training (VRBT) involves the trainee independently interacting with a computer-generated simulation of the operative room (OR). Its benefit comes from the deliberate practice of key procedural steps, which the trainee can repeat in the virtual environment before transferring these skills to real patients (Ericsson and Harwell 2019). There is widely published evidence to suggest that repetitive practice is fundamental for orthopaedic surgical training, specifically for arthroscopic surgery and key steps in trauma procedures, e.g., femoral neck guidewire placement for dynamic hip screw fixation (Mabrey et al. 2010, Sadideen et al. 2013, Stirling et al. 2014, Thomas et al. 2014, Gustafsson et al. 2019, Rölfing et al. 2020).

In line with such observations, emerging literature evidences improvements in technical aptitude from repeated VRBT (Aim et al. 2016, Bartlett et al. 2018), the eagerness of trainees to practise on the technology (Karam et al. 2013), its potential cost-effectiveness (Bridges and Diamond 1999), and its endorsement from the Royal College of Surgeons (RCS) (RCS 2019). However, in the UK access to this technology is sporadic and not equitable across training regions. Integration appears to have been stifled by a lack of urgency and inflexibility to alter the accepted apprenticeship methods of learning.

Prima facie evidence suggests a considerable reduction in the surgical training opportunities during the COVID-19 outbreak. Orthopaedic subspecialty rotations were stopped in March 2020, and trainee involvement in theatres was reduced to make way for consultant-delivered procedures while also making sure that fewer individuals were exposed to these aerosol-generating procedures. Finally, there is an ongoing postponement of elective cases. All this is occurring against a background of many emerging technically challenging techniques (e.g., arthroscopy and navigated surgery) and a reduction in theatre exposure secondary to the European Working Time Directive (Fitzgerald and Caesar 2012). Regardless of these disruptions, the Joint Committee on Surgical Training (JCST) surgical curriculum in the UK has maintained the minimum number of certain index procedures that are deemed crucial for all orthopaedic surgeons to formally complete their training.

Adaptive solutions are required to maintain the holistic development of orthopaedic registrars (Kelc et al. 2020). Compared with alternative methods of experiential, non-OR learning, which include cadaver-based dissection and physical mannikins, high-fidelity VRBT provides realistic 3D anatomy and haptic feedback to truly mimic the OR environment. Its use is flexible to the time of the trainee and cases are changeable to the learning requirements of the learner (Vaughan et al. 2016). This has been extensively referenced within the literature (Aim et al. 2016, Bartlett et al. 2018).

However, transitioning experiential learning into a virtual platform is not without its disadvantages. The instructivist pedagogy of VR, with an absence of crucial personal interactions, runs against experiential learning theories, which highlight the importance of peer and multidisciplinary learning. The loss of this sensitive interaction renders VRBT incapable of developing skills that centre on communication and analysis, key requirements of the surgical curriculum (ISCP: Intercollegiate surgical curriculum programme 2020). We must therefore consider that VRBT cannot truly fully replace the “hands on” learning of the OR but could become integrated as an adjunct to theatre learning, for example with femoral neck guidewire placement. This would be supplemented with self-reflection and reflective practice, integral aspects for the professional development of orthopaedic trainees (Cruess 2006). Altogether, given these aforementioned deficiencies, the future development of this technology could see the incorporation of “group” VRBT, whereby individuals could communicate and interact together on a case to truly mimic the multidisciplinary nature of the theatre environment.

Altogether, the future of orthopaedic teaching is evolving, and technology must be at the centre of this change. With the current relative lack of theatre time for orthopaedic trainees as a result of the COVID-19 pandemic, and the possibility of futures waves, the role of VBRT will become increasingly obvious as a safe, reproducible, adjunctive, and cost-effective way of developing and maintaining surgical training. As we emerge from this pandemic, let us not reach back to the normal, but instead reach out for the better, adapt our practices, and bring orthopaedic learning into the 21st century.

The authors declare no conflicts of interest
